# Supplementary material for: Effects of Community-Based Interventions on Medication Adherence and Hospitalization for Elderly Patients with Type 2 Diabetes at Primary Care Clinics in South Korea
Source: Int J Environ Res Public Health. 2021 Mar 25;18(7):3396. doi: 10.3390/ijerph18073396 (PMC8059144; doi:10.3390/ijerph18073396)
Supplement: Supplementary file 1 [file ijerph-18-03396-s001.pdf]

Supplementary Table 1. Comparisons of geographical characteristics between Gwangmyeong city and two control group areas in 2009

area	Population size (million)	Financial autonomy of local government budget (%)	Number of physicians per 1,000 people	Share of elderly (≤65) population in total population (%)	Portions of apartments in housing (%)
Gwangmyeong city	0.316	37.8	1.97	10.77	32.8
A city*	0.273	38.8	2.01	10.01	39.9
B city*	0.227	36.9	1.73	7.68	38.6
